# Group III metabotropic glutamate receptors: guardians against excitotoxicity in ischemic brain injury, with implications for neonatal contexts

**DOI:** 10.1007/s43440-024-00651-z

**Published:** 2024-09-17

**Authors:** Damian Mielecki, Elżbieta Salińska

**Affiliations:** Department of Neurochemistry, Mossakowski Medical Research Institute, Polish Academy of Sciences, Pawińskiego 5, Warsaw, 02-106 Poland

**Keywords:** Brain ischemia, Excitotoxicity, Group III mGluRs, Metabotropic glutamate receptor, Neonatal birth ischemia, Neonatal hypoxic-ischemic encephalopathy

## Abstract

The group III metabotropic glutamate receptors (mGluRs), comprising mGluR4, mGluR6, mGluR7, and mGluR8, offer neuroprotective potential in mitigating excitotoxicity during ischemic brain injury, particularly in neonatal contexts. They are G-protein coupled receptors that inhibit adenylyl cyclase and reduce neurotransmitter release, mainly located presynaptically and acting as autoreceptors. This review aims to examine the differential expression and function of group III mGluRs across various brain regions such as the cortex, hippocampus, and cerebellum, with a special focus on the neonatal stage of development. Glutamate excitotoxicity plays a crucial role in the pathophysiology of brain ischemia in neonates. While ionotropic glutamate receptors are traditional targets for neuroprotection, their direct inhibition often leads to severe side effects due to their critical roles in normal neurotransmission and synaptic plasticity. Group III mGluRs provide a more nuanced and potentially safer approach by modulating rather than blocking glutamatergic transmission. Their downstream signaling cascade results in the regulation of intracellular calcium levels, neuronal hyperpolarization, and reduced neurotransmitter release, effectively decreasing excitotoxic signaling without completely suppressing essential glutamatergic functions. Importantly, the neuroprotective effects of group III mGluRs extend beyond direct modulation of glutamate release influencing glial cell function, neuroinflammation, and oxidative stress, all of which contribute to secondary injury cascades in brain ischemia. This comprehensive analysis of group III mGluRs multifaceted neuroprotective potential provides valuable insights for developing novel therapeutic strategies to combat excitotoxicity in neonatal ischemic brain injury.

## Introduction

Brain ischemia, particularly in neonates, is a critical condition that leads to significant morbidity and mortality. This condition is characterized by a lack of oxygen and glucose supply to the brain, resulting in neuronal injury and excitotoxicity primarily mediated by excessive glutamate release. The role of glutamate as a neurotransmitter is crucial for efficient brain functioning in physiological conditions; however, an increase in its concentration and overactivation of ionotropic glutamatergic receptors can lead to neuronal death through excitotoxic mechanisms. In this context, metabotropic glutamate receptors (mGluRs), specifically group III mGluRs (mGluR4, mGluR6, mGluR7, and mGluR8), emerge as potential neuroprotective agents. These receptors are predominantly presynaptic and play a vital role in modulating neurotransmitter release, thereby offering a promising target for therapeutic intervention [1–3].

The rationale for this review stems from the need to better understand the neuroprotective mechanisms of group III mGluRs in the context of neonatal cerebral ischemia. While traditional neuroprotective strategies have focused on ionotropic glutamate receptors, their direct inhibition often results in adverse effects due to their essential roles in normal synaptic function and young brain development [4]. In contrast, group III mGluRs provide a more refined approach by modulating glutamatergic transmission rather than completely blocking it, potentially reducing excitotoxicity without compromising vital synaptic activity [1, 2].

This review aims to comprehensively examine the differential expression and functional roles of group III mGluRs across various brain regions, particularly emphasizing their implications in neonatal brain development and injury. We will explore the signaling pathways activated by these receptors, their influence on intracellular calcium dynamics, and their broader impact on neuroinflammation and oxidative stress in the context of excitotoxicity.

To ensure a thorough and relevant selection of literature, a systematic search strategy was employed. This involved querying databases for peer-reviewed articles focusing on group III mGluRs, excitotoxicity, and neonatal brain injury. A citation network was established by cross-referencing relevant studies, ensuring that the selected works contribute meaningfully to the understanding of the topic. By synthesizing current research, this review aims to highlight the therapeutic potential of targeting group III mGluRs in developing effective treatments for neonatal cerebral ischemia and related conditions.

## Overview of group III metabotropic glutamate receptors

Group III metabotropic glutamate receptors (mGluRs) comprise four members: mGluR4, mGluR6, mGluR7, and mGluR8. They are characterized by their coupling to G_i/o_ proteins, leading to the inhibition of adenylyl cyclase, decrease in cyclic adenosine monophosphate (cAMP) levels, and subsequent reduction in protein kinase A (PKA) activity [1]. This results in the inhibition of voltage-gated calcium channels and activation of G-protein-coupled inwardly-rectifying potassium (GIRK) channels, which hyperpolarize the neuron and reduce neurotransmitter release [5]. The affinity of glutamate for group III mGluRs varies among the different members. mGluR4 has EC_50_ for glutamate of approximately 3 - 20 µM, mGluR6 has EC_50_ of around 7 - 38 µM, mGluR7 exhibits EC_50_ values of 56 - 5400 µM, and EC50 for mGluR8 were established to be in a range of 0.02 - 11 µM [PMID: 10443583; 10530808]. Notably, mGluR7 has a significantly lower affinity for glutamate compared to the other group III receptors, which may reflect its unique role in synaptic transmission.

Group III mGluRs are primarily localized on presynaptic terminals and are involved in the inhibition of neurotransmitter release, including glutamate, GABA, and monoamines, acting as autoreceptors and/or heteroreceptors [6–11]. Their activation leads to a decrease in neuronal excitability and synaptic transmission, indicating acting as negative feedback regulators of glutamatergic neurotransmission. This contrasts with group I mGluRs, which generally enhance neuronal excitability and synaptic transmission. Group III mGluRs have been shown to modulate long-term potentiation (LTP) induction in various brain regions, including the hippocampus and amygdala. These findings highlight the complex and region-specific roles of group III mGluRs in modulating LTP induction and maintenance, acting as either facilitators or inhibitors of LTP depending on the brain region and specific synaptic connections involved [5, 12–14]. Additionally, group III mGluR inhibition-mediated plasticity in the CA2 region involves ERK/MAPK signaling and downregulation of STEP protein [5].

The group III mGluRs modulated pathways are crucial for the neuromodulatory roles of group III mGluRs in various physiological and pathological processes, including neuroprotection, pain modulation, and the regulation of synaptic plasticity [1, 15, 16]. Compared to group II mGluRs (mGluR2 and mGluR3) which are primarily located postsynaptically and mediate a fast onset of inhibitory neurotransmission, potentially acting as low-pass filters, group III metabotropic glutamate receptors are predominantly presynaptic (except for mGLuR6) and cause a slower onset of neurotransmitter release inhibition. Activation of group II mGluRs rapidly enhances paired-pulse facilitation, indicative of a presynaptic mechanism, whereas group III mGluRs differentially regulate GABAergic synaptic transmission to distinct GABAergic interneurons [5, 17–20]. This presynaptic localization and ability to inhibit neurotransmitter release make group III mGluRs particularly important in the context of brain ischemia, where a surge of glutamate release can lead to excitotoxicity and neuronal death. The mechanism of action is strictly linked to the subcellular localization. One of the main routes of action of group III mGluRs is to inhibit the NMDA receptors. These receptors are localized on synaptic or extrasynaptic membranes. In hypoxic-ischemic conditions, activation of the synaptic N-methyl-D-aspartate receptors (NMDARs) supports defense processes and is beneficial for neuronal survival. The extrasynaptic NMDARs, activated by excessive glutamate release, trigger an intracellular cascade leading to apoptosis by interacting with the transient receptor potential cation channel subfamily M member 4 (TRPM4). The ability of group III mGluRs to inhibit the activation of extrasynaptic NMDARs creates conditions for stopping the neurodegenerative processes that accompany brain ischemia [4, 21].

## Distribution of group III metabotropic glutamate receptors in the brain

**Fig. 1:**
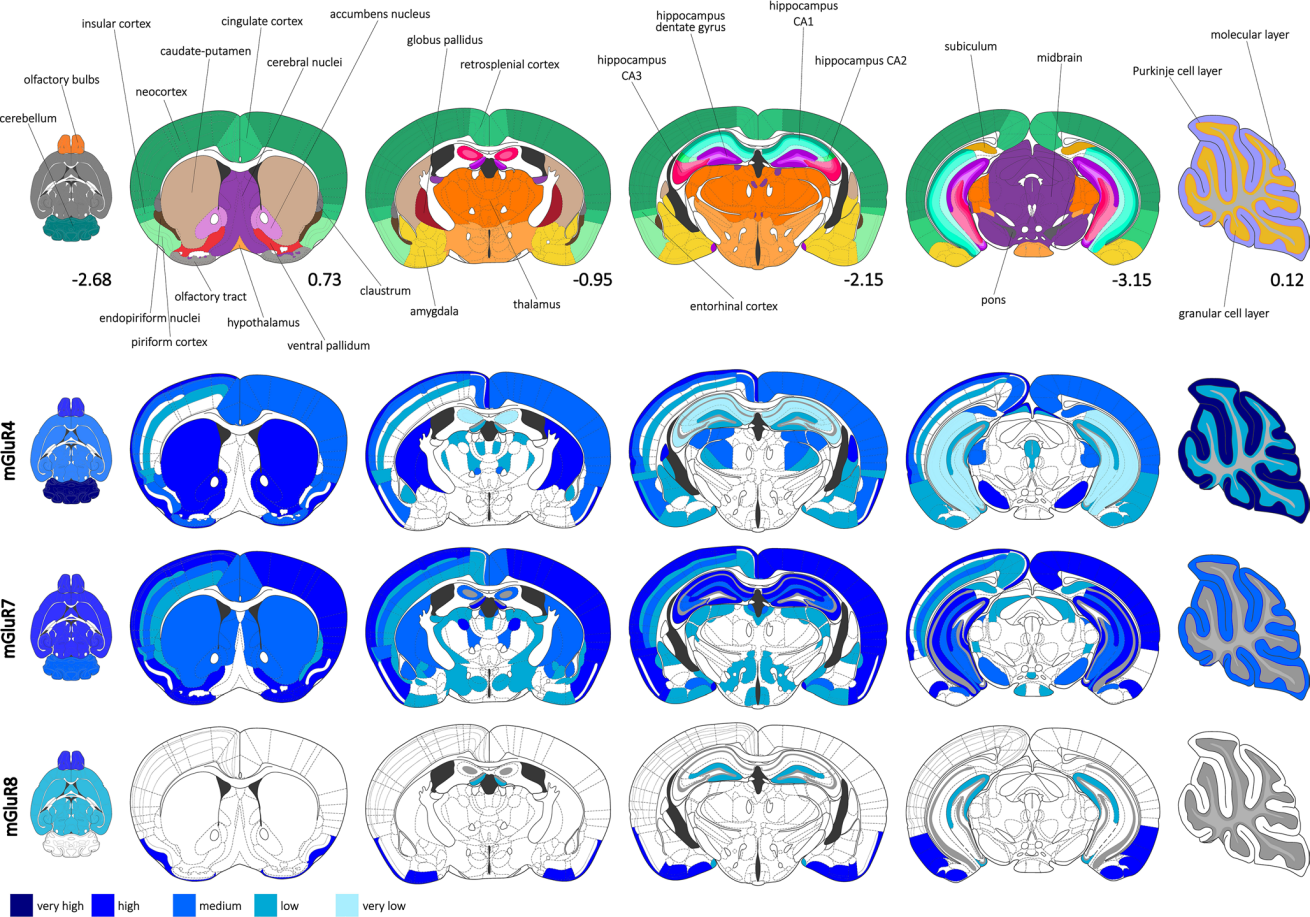
Expression of group III mGluRs, mGluR4, mGluR7, and mGluR8, in the brain, based on immunocytochemical data. These three mGluRs are generally widely expressed in the central nervous system. The first panel shows the localization of main brain compartments, color-coded, with one ventral, four coronal, and one sagittal sections. In the coronal sections, the left hemisphere shows the cortex divided into layers the right hemisphere shows the averaged intensities of the immunocytochemical staining signals (distance from bregma, or midline in the case of sagittal section of the cerebellum, marked below the figures). Panels 2–4 present expression of mGluR4/7/8 in different brain regions. The relative estimated protein concentrationis coded by the hues of blue. The brain frames are based on the Paxinos and Franklin’s *The Mouse Brain in Stereotaxic Coordinates*, where detailed description of indicated structures can be found [145, 146]. The figure was prepared in Inkscape 1.3.2.

It is known that group III mGluRs, unlike group II mGluRs, are broadly distributed in the brain and peripheral organs. The locations where the receptor is highly or minimally present are closely linked to its function and the potential pharmacological applications of compounds that can influence its activity. Research has revealed the widespread expression of group III mGluRs in the central nervous system, with certain distinct features depending on the specific mGlu receptor and the model utilized, whether it be the mouse or rat brain (Fig. 1).

mGluR4/mGluR4a is expressed in particular regions of the brain and generally confined to neuronal cells. The expression of its gene was predominant and characteristic of the cerebellum. It was also most commonly detected in the olfactory bulb (granule and periglomerular cells), thalamus, lateral septum, olfactory tubercule, and pontine nucleus [22–25]. The mRNA of mGluR4 could also be found in the intercalated nuclei of the amygdala, medial mammillary nucleus, zona incerta, superior colliculus, dorsal cochlear nucleus, and some other regions of lower brainstem, like periaqueductal gray and interpeduncular nucleus [25]. The cortical regions did not express mGluR4 at high levels but it could be found in the entorhinal cortex (inner part), subicular regions, and neocortex (layers III, IV, and V) [23, 25, 27]. In the cerebellum, the strongest expression was seen in granule and, to a lesser extent, in Golgi cells [22, 24, 25]. In the hippocampus, the mRNA of this receptor was quite abundant in the pyramidal cells of CA2, with detectable quantities in pyramidal cells of CA3 and granule cell layer of the dentate gyrus, and quite low in pyramidal cells of CA1 [23, 25, 27]. Although mGluR4/mGluR4a expression was limited in the basal ganglia, it was found in specialized neurons, especially in the ventrolateral and ventromedial thalamus and, to a slightly lesser extent, in the nucleus accumbens, striatum, parafasicular nucleus, premotor cortex, and subthalamic nucleus, with minimal presence in other structures [22, 23]. Although most observations have been conducted on animal brains, studies on human brain sections have confirmed similar expression patterns [28].

The mGluR4a protein localization experiments were performed mainly with rats and mice. As expected, the receptor distribution can substantially differ from in situ hybridization results. The strongest signals were observed for the cerebellum but this time in the neutropil of the molecular layer, especially parallel fibers, with weak staining in the granule cell layer [26, 29–31]. Other regions with quite strong protein presence were identified as the olfactory bulb and olfactory tubercule, basal ganglia, like striatum and globus pallidus, superficial gray of superior colliculus, and particular nuclei of thalamus, especially relay nuclei, posterior thalamic nuclei, mediodorsal thalamic nucleus (central part), lateral posterior thalamic nucleus, ventral posterolateral thalamic nucleus, and lateral dorsal thalamic nucleus. Only layer I of the neocortex was highly stained, with layers II, III, and V showing moderate protein levels. Quite low protein presence was observed in e.g. amygdalar nuclei (lateral and basolateral) and some other thalamic nuclei [26]. In the hippocampal regions, relatively high levels of mGluR4a were observed only in the inner third of the molecular layer of the dentate gyrus, constituting a primary terminal zone of the associational/commissural pathway, with very little staining in terminal fields of the perforant path. The additional presence of mGluR4 was also detected in dendritic shafts of interneurons in the hilus and molecular layer of the dentate gyrus, thus probably contributing to inhibiting the excitatory GABAergic synapses, and in type II dendritic profiles of interneurons in stratum oriens/alveus of CA1, including somatodendritic profiles of interneurons expressing mGluR1a [26, 32, 33].

Worth mentioning, mGluR4 can act as a presynaptic autoreceptor in synapses located in the cerebellar cortex, substantia nigra pars reticulata, and striatum; however, in projections of GABAergic neurons from striatum and nucleus accumbens, it can function as heteroreceptor in globus pallidus, ventral pallidum, substantia nigra pars reticulata, and entopeduncular nucleus [26, 29, 31, 34], corroborated by electrophysiological studies [11].

mGluR4a is often co-localized with N- and P/Q-type calcium channels at presynaptic active zones [38]. This close spatial arrangement allows for tight coupling between the receptors and calcium channels, enabling efficient modulation of channel function by the receptors, potentially leading to a broader modulation of synaptic activity [39]. Activation of mGluR4a can inhibit these presynaptic calcium channels, leading to reduced calcium influx upon neuronal depolarization [40]. This, in turn, decreases the probability of synaptic vesicle exocytosis and neurotransmitter release at the synapse. The ability of mGluR4a to affect multiple calcium channels may provide a more generalized inhibitory effect on synaptic transmission, which could be crucial in regulating excitability and preventing excessive neurotransmitter release [40]. The inhibition of N- and P/Q-type channels is essential, as these channels are key players in neurotransmitter release at many synapses, and their modulation can significantly impact synaptic dynamics and neuronal excitability [41, 42].

The expression of mGluR4 increased with postnatal development, as analyzed in postnatal days P1 to P30 [43]. The main source of this phenomenon was the substantial increase in the expression of the receptor in the cerebellum, starting from merely detectable on P3. On the other hand, the relatively high levels of mGluR4 mRNA in the thalamus and septal nuclei decreased during the development of rats, and its level in the striatum remained quite low but constant [43]. The immunohistochemical studies conducted in rats corroborated these observations indicating the strong signal of mGluR4a in cell bodies of cortex layers II– VI deteriorating starting from P12 [44]. In the striatum, the first significant level of mGluR4a observed in cell bodies and fibers at P12, consequently migrated to the striatal projections to the substantia nigra pars reticulate [44]. The considerable signal in the cellular layers of the hippocampus remained quite constant up to P60, and additional staining in fibres, as described above, appeared in stratum oriens [44].

The mGluR6 receptor shares the highest sequence similarity with the mGluR4a subtype. It is exclusively expressed in the postsynaptic membranes of ON-bipolar cells within the retina, where it plays a crucial role in the transduction of visual signals [45]. It was not found in the brain, consequently, its further description will be omitted from the following sections.

The mGluR7 receptor is the most widely expressed member of the group III metabotropic glutamate receptor family, comparably to mGluR4, in neuronal cells. Particularly high levels of its mRNA were observed in the cerebral cortex (especially occipital, primary olfactory, retrosplenial, striate, and layer I of piriform cortex), olfactory bulb (mitral and tufted cells), olfactory tubercule, islands of Calleja, hippocampus, superior and inferior colliculi, striatum, medial septal nucleus, thalamus (especially anterodorsal, central medial, and paraventricular nuclei), hypothalamus (particularly periventricular zone and medial mammillary nucleus), amygdala (especially nucleus of the lateral olfactory tract and the bed nucleus of the stria terminalis), locus ceruleus, nucleus of the solitary tract, and dorsal root and trigeminal ganglia [23, 25, 46–49]. Layers II to V of neocortex showed the high levels of mGluR7mRNA, with layer IV predominating [23, 47, 50]. In the cerebellum, the expression of mGluR7 was relatively low overall and found mainly in Purkinje cells with some expression in Golgi cells [25, 46, 47, 50]. In the hippocampus, the granular layer and pyramidal layers of CA2 and CA3, with a little bit less staining in CA1, produced especially significant levels of mRNA, with some expression in the hilus [23, 25, 46, 47, 49, 50]. The expression of mGluR7a mRNA was abundant as compared to the mGluR7b, notwithstanding the striatum with equal synthesis of both mRNAs, and olfactory bulb with the absence of mGluR7b mRNA [49]. In basal ganglia, mGluR7a/mGluR7b predominated in the premotor cortex and nucleus accumbens, with significant expression in the striatum, and quite considerable in ventrolateral and ventromedial thalamus, and subthalamic nucleus [23, 50].

Kinoshita and co-workers performed a very detailed analysis of protein localization for both the splice variants, mGluR7a and mGlur7b, with other work focusing on the presence of mGLuR7a in basal ganglia [50, 51]. Generally, the mGluR7a variant was much more frequent and widely distributed in all brain regions. Some of the areas with especially high levels of mGluR7a included the olfactory bulb (glomerular and internal plexiform layer in main olfactory bulb and external plexiform, mitral, and granule cells in accessory olfactory bulb), olfactory tubercule, islands of Calleja, cerebral cortex (including especially layer I of neocortex, piriform, and entorhinal cortex), hippocampus, and amygdala (especially periamygdaloid cortex and amygdalohippocampal area) [50, 51]. In the thalamus, hypothalamus, and midbrain, the mGluR7a was localized in particular nuclei. In the thalamus, these included the anteroventral nucleus, nucleus of the stria medullaris, mediocaudal part of the lateroposterior nucleus, dorsal lateral geniculate nucleus, and magnocellular part of the ventral lateral geniculate nucleus. In the hypothalamus, the median part of the medial mammillary nucleus and supramammillary nucleus showed relatively high signals for mGluR7a protein. The zonal and superficial gray layers of the superior colliculus, as well as locus ceruleus, caudal subnucleus of the spinal trigeminal nucleus, and lamina of the spinal cord were other areas with strong mGluR7a signal [50, 51]. The splice variant, mGLuR7b, longer by 7 amino acids at the C-terminus, was present in fewer brain structures than mGluR7a, being mainly the islands of Calleja, ventral pallidum, globus pallidus, substantia innominata, substantia nigra pars reticulata, lateral part of the bed nucleus of the stria terminalis, with overall low levels in cerebral cortex. In the cerebellum, only the Purkinje cells showed quite elevated levels of mGluR7, with molecular layer reaching moderate protein presence, but it was revealed that the only source of immunological signal was the mGluR7b splice variant reaching additionally high levels in cerebellar nuclei [50–52]. As mentioned earlier, the hippocampus showed quite high levels of the mGluR7 receptor, with the predominance of mGluR7a in the stratum lacunosum moleculare of CA3 (especially the inner layer), all stratum oriens CA regions, stratum radiatum of CA1, stratum lacunosum moleculare of CA2, and stratum moleculare of the dentate gyrus (especially middle one third, termination of perforant path fibers and dendrities of interneurons from hilus or granule cell layer) but the cellular layers, moderate presence in stratum lacunosum moleculare of CA1, stratum radiatum of CA2 and CA3, and the hilus of the dentate gyrus (somatic and dendritic profiles), and low in stratum lucidum of CA3. The somatic and dendritic profiles were also stained at the stratum oriens-alveus border of CA1. High presence of mGluR7b was found only in the hilus of dentate gyrus, and stratum lucidum of CA3. Generally, mGluR7 localized at synapses of non-mossy fibers, mostly presynaptically, but with sporadic postsynaptic localization [32, 37, 51]. Worth mentioning is the fact that in the hippocampal interneurons, most prominently in the border zone of the stratum oriens/alveus of CA1, dendrites of interneurons expressing mGluR1a were making synapses with mGluR7a and mGluR7b [32, 53]. Mouse brain was lacking mGluR7a in the medial habenular nucleus in comparison to rat [51].

Interestingly, as stated by Kinzie and co-workers (1995), the mGluR7 can participate in specific neurotransmitter pathways in the caudate-putamen since its immunostaining in this part of the brain was irregular [47]. mGluR7a was localized to presynaptic membranes in the basal ganglia, where it functions as an autoreceptor, while also being present in postsynaptic terminals of striatopallidal and striatonigral projections, regulating GABA release [23, 50, 54].

The mGluR7 receptors are co-localized with N-type calcium channels and selectively reduce their activity [38, 55]. N-type channels are known to play a crucial role in regulating neurotransmitter release at many central synapses [41, 42]. The consequences of this selective inhibition are distinct from those of mGluR4a. When mGluR7 is activated, it primarily reduces the activity of N-type calcium channels without affecting P/Q-type channels. This specificity allows for fine-tuning of synaptic transmission at certain synapses where N-type channels are predominant. The selective modulation by mGluR7 can lead to a more precise control of neurotransmitter release, which may be essential for specific forms of synaptic plasticity and signal processing in neural circuits [40].

The developing rat cerebellum exhibited high levels of mGluR7 expression as early as embryonic day 18 (E18). A similar phenomenon was observed in the granule cells of the olfactory bulb, where the expression in the cells of the glomerular layer only began at postnatal day 7 (P7). Additionally, the synthesis of mGluR7 in the neocortex peaked at birth (P0). Intriguing changes also occurred in the hippocampus, where the expression of mGluR7 was more pronounced in the CA1-CA3 regions at birth before the proportions between the CA regions and the dentate gyrus shifted sequentially [47].

The mGluR8 receptor, as compared to the mGluR4 and mGluR7, is expressed in the brain at a relatively low level. It also exists in two splice variants, mGluR8a and mGluR8b. Its mRNA was mainly synthesized in the olfactory bulb (mitral, granule, and periglomerular cell layers), pontine nuclei (especially reticulotegmental nucleus), reticulate nucleus, especially lateral reticulate nucleus of the thalamus, and piriform cortex (superficial layer and layer II). The mGluR8 mRNA could be also found in the cerebral cortex (deep layers), hippocampus, cerebellum, mammillary body, basal amygdaloid nucleus, periaqueductal gray, septum, superficial layers of superior colliculus, and other layers of isocortex and in hindbrain [57, 58]. In all these regions, the main source of the signal came from the mGluR8a mRNA, with the exception of the cortex and hippocampus, where the signal from mGluR8b mRNA was equal, and the lateral reticular, ambiguous, and the spinal vestibular nuclei with only mGluR8a mRNA [49]. As expected, in the hippocampus, only the pyramidal cell layers of CA1-Ca3 and the granule cell layer of the dentate gyrus were positively stained for the mGluR8 mRNA [57]. Expression of mGluR8 in the cerebellum was relatively low, with the receptor found in a subpopulation of stellate/basket and Golgi cells in the molecular layer [35]. Considering the basal ganglia motor loop, the mGluR8a/mGluR8b was found at the highest levels in the reticular thalamic nucleus and premotor cortex, and at moderate levels in the nucleus accumbens, striatum, subthalamic nucleus, substantia nigra pars compacta, parafascicular nucleus, and anteroventral thalamus (ventral anterior nucleus) [23].

The immunohistochemical staining of mGluR8 confirmed the expression studies, establishing the protein presence mainly in rhinencephalic areas, being olfactory bulb (plexiform layers of the main olfactory bulb and mitral cell layer of the accessory olfactory bulb), anterior olfactory nucleus, superficial layers of the olfactory tubercule, and layer Ia of the piriform and entorhinal cortex. The staining could be also seen in the periamygdaloid cortical regions, like the amygdalopiriform transition area [59]. In the hippocampus, mGluR8 (antibodies against C-terminal 886–908 amino acids) was localized mainly to the terminal zone of the perforant path in the neuropil of the outer third of the dentate molecular layer and the outer stratum lacunosum-moleculare of CA3. The molecular layer of the dentate gyrus was also marked with dendritic profiles of interneurons originating from the hilus and granule cell layer [32, 60].

Duvoisin and co-workers (1995) also analyzedanalyzed the expression of mGluR8 in developing mice [58]. Surprisingly, at the E16 stage, the mRNA signal was much more common as compared to the adult brain and found in the olfactory bulb, parts of the telencephalon, thalamus, hypothalamus, midbrain, pons, medulla oblongata, dorsal root and trigeminal ganglia, and retina [58].

## Glutamate surge during brain ischemia

It was established early on that one of the primary consequences of cerebral ischemia is the massive release of glutamate into the extracellular space. For instance, during transient complete brain ischemia in rats, the extracellular glutamate concentration in the hippocampus increased eightfold compared to the pre-ischemic level [61]. The glutamate leakage in the rat hippocampal slices suffering from energy deprivation reached 0.9 µM around the NMDARs [62]. A similar phenomenon was observed in the rat striatum, with a biphasic glutamate release pattern - the first phase saw a sixfold increase within the initial 10 min, followed by an additional 2.3-fold increase over the next hour, reaching a steady state [63]. Interestingly, only the first phase was dependent on the presence of calcium [63]. Conversely, the use of voltage-dependent calcium channel blockers inhibited glutamate secretion in an oxygen-glucose deprivation (OGD) model of neonatal rat cerebrocortical slice cultures, which also established that the main source of glutamate was the vesicular release [64]. On the contrary, Rossi and co-workers found that the main source of extracellular glutamate released by rat hippocampal slices, as receptor-gated current in CA1 area in conditions simulating severe ischemia, was the reversed action of glutamate transporters, and not calcium-dependent exocytosis [65]. Considering this phenomenon, research results seem to strongly depend on the particular experimental setup. The measurements of the release of glutamate based of the NMDAR-mediated current showed that the first source of increased external glutamate can be attributed to decreased net glutamate uptake, at the level of about 50%. The remaining source comes from the action potential-independent vesicular release [62]. The excitatory amino acid transporters (EAAT) were mainly responsible for the impaired glutamate uptake that took place within first few minutes of energy deprivation. These observations were valid either for CA1 or CA3 regions of rat hippocampal slices [62]. Clearly, the mixed cortical cultures derived from mice lacking mGluR4 secreted about 4.7-fold more glutamate than the cultures obtained from wild-type mice, with basal concentration to be 57 nM. Still, the use of L-2-Amino-4-phosphonobutyric acid (L-AP4), a non-specific agonist for the group III mGluRs, decreased the glutamate by about 1.6-fold, indicating the involvement of other group III mGluRs [66].

The discharge of glutamate is not dependent on the method used to induce brain ischemia, as the application of endothelin-1 in rats caused a similar effect, increasing striatal glutamate levels up to 55-fold within the first hour, along with substantial increases in dopamine and GABA [67]. A 60-fold increase in glutamate was also observed in cerebellar granule neurons [56]. However, other studies reported more modest increases, such as 1.8-fold in the hippocampus of kainate (KA)-treated rats [68] and 2.4-fold in mouse cortical neuronal cultures subjected to OGD [69]. Interestingly, the magnitude of extracellular glutamate release can vary depending on the rat strain used, with Wistar rats exhibiting higher striatal glutamate (and aspartate) levels compared to F344 rats, despite a slower rate of increase, and smaller cortical infarction volumes, although the striatal damage was comparable [70].

Glutamate and aspartate belong to the class of endogenous excitatory amino acids (EAAs). Interestingly, normally operating transporters can tackle with glutamate concentrations as low as 2 nM [71]. On the other hand, concentrations of glutamate at the level of about 1 µM can lead to death of about 50% of the cultured cerebral cortex neurons [72]. During cerebral ischemia, the concentrations of these amino acids can increase dramatically, due to induced release from neurons, reduced reuptake by neurons and glia, and the discharge from disintegrated cells, and they turn from neurotransmitters into neurotoxins [73]. Collectively, these sources contribute to a “vicious cycle” that leads to excitotoxicity, which is one of the earliest, if not the first, pathophysiological consequences of brain ischemia [70, 74]. This surge in extracellular glutamate is a key factor that makes group III mGluRs, which inhibit neurotransmitter release, an important potential therapeutic target in the context of ischemic brain injury.

The affinity of glutamate for group III mGluRs and their presynaptic localization make them particularly relevant in the context of brain ischemia. During ischemia, a surge of glutamate release can lead to excitotoxicity and neuronal death. The activation of group III mGluRs, which inhibit neurotransmitter release, may serve as a neuroprotective mechanism to counteract this glutamate-mediated excitotoxicity. This neuroprotective effect may be particularly important in the developing brain, where group III mGluRs have been shown to play a more prominent role in regulating excitotoxicity compared to the adult brain.

## Group III metabotropic glutamate receptors counteract glutamate excitotoxicity in neurons

The simplest experimental setup to study the influence of metabotropic glutamate receptors on cell survival is to induce excitotoxicity through the activating action of N-methyl-D-aspartate (NMDA) or KA and the use of broad and/or narrow specificity agonists and antagonists of mGluRs. It was established quite early that the activation of mGluRs of different types can have neurotoxic or neuroprotective action, such as the use of trans-1-aminocyclopentane-1,3-dicarboxylic acid (t-ACPD), an agonist of all mGluR groups. It is also influenced by the fact that the specific subtypes can be expressed at one pre- and/or postsynaptic membrane.

Since glutamate accumulates in the synaptic cleft after cerebral ischemia, the use of agonists of group III mGluRs for the treatment of excitotoxic effect seemed very promising. For example, non-specific agonists of group III mGluRs, L-AP4 and L-serine-O-phosphate (L-SOP), had a strong protective effect against excitotoxicity induced by NMDA or KA in cultured cortical neurons, reducing the cellular death to about 37% (lactate dehydrogenase (LDH) release was decreased to 55%) [76] [PMID: 8528465].

The authors also showed the immunoreactivity of the cultured cortical neurons to the mGluR4a [76]. Further research, including the use of non-specific agonists of group III mGLuRs ((+)-4-phosphonophenylglycine ((+)-PPG), L-AP4, and L-SOP) and cortical cell cultures from mice lacking the gene coding for mGLuR4, clearly established the involvement of mGLuR4 in the neuroprotection against the excitotoxic effect induced by NMDA [66]. The (+)-PPG, L-AP4, and L-SOP reduced the neuronal degeneration down to 20%, depending on the compound and concentration used, with no effect in mixed cortical cultures derived from knock-out mice [66]. Similar phenomena were observed in the case of rats with the infusion of NMDA directly into the striatum and rats treated with (RS)-PPG when quinolinic acid was used instead of NMDA, significantly reducing the infarct volume [77].

In the cerebellum, contrary to the cerebral cortex, the main anti-excitotoxic activity can be ascribed to mGluR7, as shown through the use of non-specific agonists, L-AP4 and L-SOP [56]. Although all three group III mGluRs are expressed in the brain, Lafon-Cazal and co-workers (1999) used the fact that low concentrations of L-AP4 (up to 100 µM) are sufficient to activate mGluR4 and mGluR8, but did not observe a neuroprotective effect in this case. On the other hand, increasing the concentration of L-AP4 (> 1 mM) did induce neuroprotection by about 35 and up to 59% for 3 and 6 mM L-AP4, respectively (LDH was reduced by 12 and 21%) [54, 56]. This is because mGluR7 presents low affinity to L-AP4 [46]. Moreover, the protective activity of mGluR7 was independent of somatic voltage-dependent calcium channels [56].

The type of neurons should be taken into consideration when assessing the potential neuroprotective activity of mGluRs against excitotoxicity. In the case of mouse striatal neuronal cultures, which are enriched in GABAergic neurons, L-AP4, and L-SOP, in concentrations activating mGluR7 caused the opposite effect, exacerbating the toxic impact of NMDA by 2-fold when used in 1 mM concentration. Similar detrimental effects were also seen in control conditions [54, 78]. This suggests that the activation of mGluR7 in GABAergic neurons can have a damaging outcome. Not surprisingly, baclofen, an agonist of GABA_B_ receptor, also intensified the toxic effect of NMDA in these cultures. Measurements of glutamate and GABA concentrations provided further insights. The presence of L-AP4, alongside the stimulation by NMDA, additionally increased the glutamate concentration by 23–29%, depending on the NMDA concentration use, and by 33% in the presence of an antagonist of NMDAR, (5S,10R)-(+)-5-Methyl-10,11-dihydro-5H-dibenzo[a,d]cyclohepten-5,10-imine hydrogen maleate (MK-801). However, the GABA concentration was decreased by 2-fold (2.7-fold in the control condition with MK-801) [54]. These findings indicate that the neuroprotective or neurotoxic effects of mGluR activation can vary depending on the specific neuronal population and neurotransmitter systems involved.

In real-life situations, the treatment of brain ischemia or stroke, whether neonatal or occurring in adulthood, can only take place after the incident. Therefore, it is important to consider this aspect in the setup of in vitro experiments. There are only a few reports on this subject, sometimes with opposite results. For example, the stimulation of mGluR7 with L-AP4 only after the induction of neurotoxicity by NMDA did not generate neuroprotective results in mouse cerebellar granule neurons [56]. On the other hand, it was shown that (1S,3R,4S)-1-aminocyclopentane-1,3,4-tricarboxylic acid (ACPT-I) (at concentrations up to 200 µM), a general agonist of group III mGluRs, inhibited the caspase-3 activity and apoptotic cell death in in vitro cultures of mouse cortical and hippocampal neurons, even when added 3 h after the excitotoxicity induction with KA. Surprisingly, the compound was much more effective in the case of hippocampal neurons, decreasing the LDH release by even 60%. It has been shown that hippocampal neurons were slightly more sensitive, by about 7%, than cortical ones. However, the induction of mGluRs of group III with ACPT-I, even 1 h after the KA treatment, proved to be very protective, restoring the viability to almost 81% for hippocampal cells and 95% for cortical neurons [68].

Similar results were seen in the KA-induced pyramidal cells damage in the rat hippocampus, where the ACPT-I, applied 30 min after KA, restored the cell viability up to 63%. A higher quantity of the compound was necessary to show an effect when injected 1–3 h after the insult [68].

The mGluR7 is particularly engaged in restoring neuronal viability after the KA-induced neurotoxicity. The N,N′-dibenzhydrylethane-1,2-diamine (AMN082), a potent allosteric agonist of mGluR7, increased the survival of mouse cortical and hippocampal neurons by 10–27% and 18–37%, respectively, depending on the concentration of the compound and time of application (30 min–1 h). These results were confirmed by the measurements of LDH release, ranging 21–37% and 29–40%, respectively. The main route of action was established to be by reducing the activity of caspase-3. Importantly, the beneficial effect was more profound in the case of hippocampal culture [79].

When investigating the role of glutamate in excitotoxicity and ischemic brain damage, as well as the role of mGluRs, it is crucial to assess and, if necessary, investigate the differences in the suite of receptors active at the synaptic cleft, and related processes, between neonates and adults.

Hilton and co-workers (2006) showed that, although glutamate was released after ischemic brain damage in animals being at the developmental stage corresponding to human infants, the glutamate caused a much more potent intracellular calcium upsurge compared to KA. Specifically, the calcium increase was 7.7-fold higher at 10 µM glutamate and 2.7-fold higher with KA, and the reaction was much faster [80].

The underlying source of this phenomenon is the fact that neonatal animals, specifically rats, did not respond to NMDA, 2-amino-3-(5-methyl-3-oxo-1,2-oxazol-4-yl)propanoic acid (AMPA), or KA treatment to the same extent as adult ones [81–84]. Moreover, the effect of intracellular calcium release stimulated by glutamate was mediated by mGluRs of group I and III, as the use of (RS)-1-Aminoindan-1,5-dicarboxylic acid (AIDA) and (RS)-α-methylserine-O-phosphate (MSOP), antagonists of these receptors, decreased the effect by about 40 and 60%, respectively. On the other hand, the group III mGluR agonists, DCG-IV and L-AP4, did not change the intracellular calcium concentration, in contrast to (S)-3,5-dihydroxyphenylglycine (DHPG), an agonist of group I mGluRs, which increased it to about 75% of the sole glutamate treatment. The data suggested that group I mGluRs are mainly responsible for the observed effect [80].

These findings highlight the importance of considering the developmental stage-specific differences in glutamate receptor expression and function when studying excitotoxicity and neuroprotection in the context of ischemic brain injury.

## Activation of group III metabotropic glutamate receptors protects neurons from ischemia-induced death

**Fig. 2:**
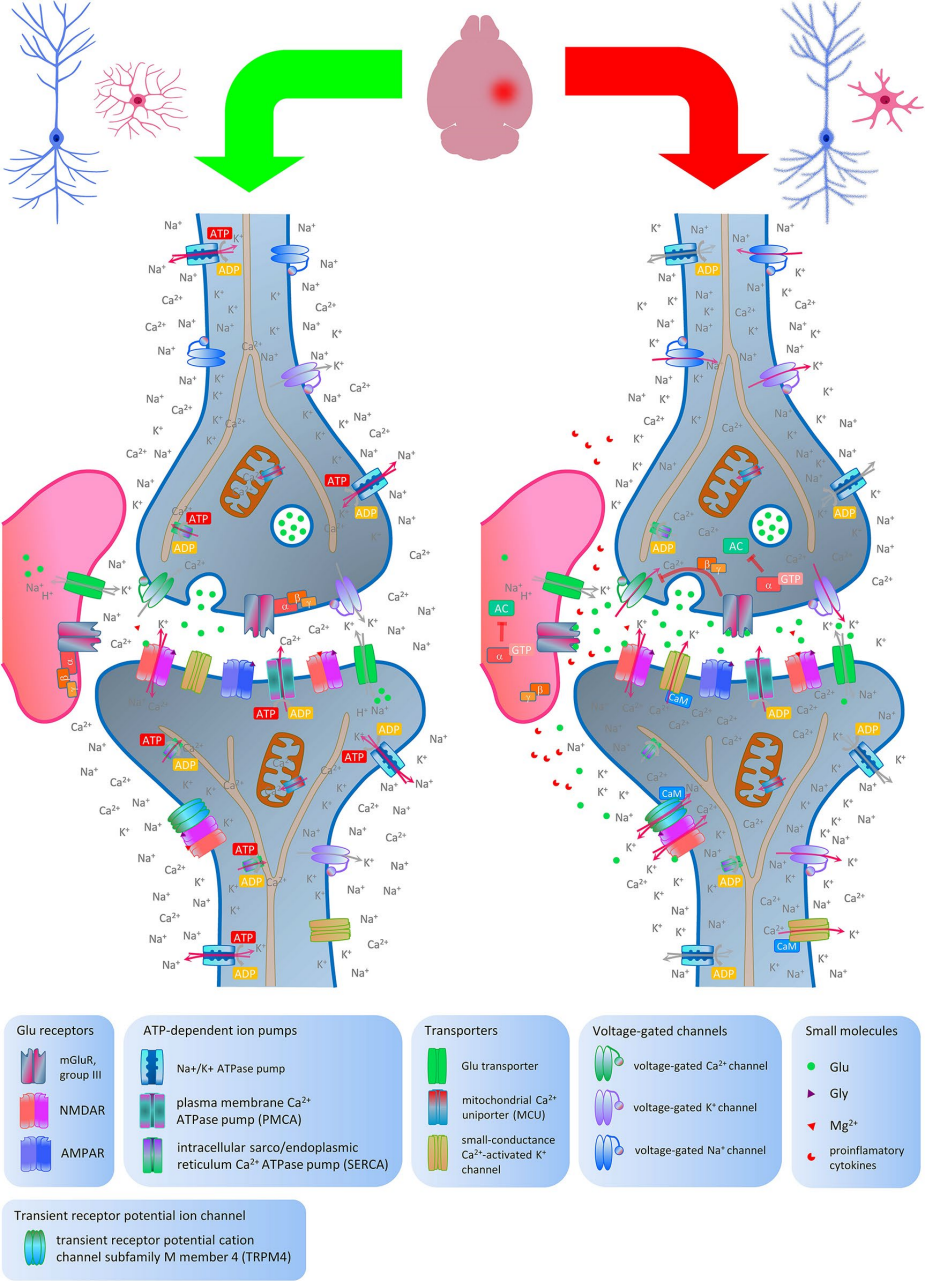
Physiological and pathophysiological states of neurons, emphasizing the protective role of group III metabotropic glutamate receptors (mGluR4, mGluR7, mGluR8), located mainly in presynaptic membrane, following brain ischemic event. Under homeostatic conditions, the resting membrane potential is actively maintained by the Na^+^/K^+^ ATPase pump. The low cytosolic Ca^2+^ concentration is regulated by three key mechanisms: the plasma membrane Ca^2+^ ATPase pump (PMCA), the sarco/endoplasmic reticulum Ca2 + ATPase pump (SERCA), and the mitochondrial Ca^2+^ uniporter (MCU), which relies on the mitochondrial membrane potential generated by ATP hydrolysis. Glutamate is released into the synaptic cleft, where it activates receptors such as N-methyl-D-aspartate receptors (NMDARs), allowing Ca^2+^ ions to enter the cell. NMDAR activation also requires glycine and the removal of Mg^2+^ by action potentials. Glutamate is primarily cleared by transporters in the postsynaptic membrane, astrocytes, and microglia. During ischemia, several pathological events occur, including the activation of microglial cells. (1) ATP-dependent pumps cease functioning, leading to membrane depolarization and intracellular Ca^2+^ accumulation. (2) Depolarization activates voltage-gated channels, disrupting the Na^+^/K^+^ gradient and further increasing intracellular Ca^2+^ levels. Glutamate transporters begin to release glutamate into the synaptic cleft, and voltage-gated Ca^2+^ channels in the presynaptic membrane trigger vesicular secretion. (3) The excess of glutamate starts to leak into extrasynaptic cleft. Overactivation of NMDAR and α-amino-3-hydroxy-5-methyl-4-isoxazolepropionic acid receptor (AMPAR) ionotropic glutamate receptors in the postsynaptic membrane results in excessive Ca^2+^ influx into dendritic synapses, potentially leading to apoptosis; however, the extrasynaptic NMDARs are rather involved in glutamate excitotoxicity. When activated, they cause an excessive influx of Ca^2+^ into the cellular body of a neuron. Their induction leads to the activation of the transient receptor potential cation channel subfamily M member 4 (TRPM4), further causing membrane depolarization. Overall, these processes trigger the signaling cascade leading to apoptosis. (4) Stimulated astrocytes and microglia secrete proinflamatory cytokines. In contrast, cells tackle the deleterious overstimulation at several levels. The small-conductance Ca^2+^-activated K^+^ channel can lead to hyperpolarization by allowing the K^+^ efflux. The activated group III mGluRs mitigate the harmful effects of excessive glutamate by inducing several processes. (1) They scavenge glutamate. (2) The group III mGluRs downstream signaling pathways, primarily involving G_i/o_ proteins, are initiated. The G(βγ) subunit can inhibit voltage-gated Ca^2+^ channels and help restore the resting membrane potential. The figure was prepared in Microsoft PowerPoint 2013 and Inkscape 1.3.2

Excitotoxicity is one of the primary contributors to neuronal and other cell deterioration following cerebral ischemia. However, this pathological process is multifaceted and involves energy failure, free radical formation, inflammation, and apoptosis. The effects of modulating mGluRs activity on the outcome of cerebral ischemia can be directly assessed in various models, including in vitro OGD or transient/permanent focal/global brain ischemia. The overall role of group III mGluRs in pathophysiology of cerebral ischemia is depicted in Fig. 2.

In rat brains subjected to transient cerebral ischemia, the expression of mGluR4, among others, significantly increased in the hippocampus and cortex, with a slight decrease in the ventral posteromedial thalamic nucleus, 24 h after insult [85, 86]. The most pronounced changes were observed for the mGluR4b splice variant [86].

The hippocampus is one of the brain regions most vulnerable to ischemic injury. However, the activity of rat hippocampal slices was restored up to 88% by post-event treatment with (RS)-PPG, a potent agonist of group III mGluRs [77, 87]. This compound was used at a relatively low concentration of 30 µM, which would allow for the activation of either mGluR4 or mGluR8, or both, but not mGluR7, as the EC_50_ values were established to be 5.2 ± 0.7, 0.2 ± 0.1, and 185 ± 42 µM, respectively [88].

In contrast, the treatment of gerbils and rats with (RS)-PPG followed by the induction of global cerebral ischemia, did not recover hippocampal neurons, and higher concentrations of the compound resulted in animal deaths. Similarly, the treatment of mice subjected to middle cerebral artery occlusion with this agonist did not improve the outcome [77]. Interestingly, the application of (RS)-PPG resulted in a reduction in infarct size in rats injected with quinolinic acid directly into the striatum [77].

Post-event treatment of in vitro and in vivo models of cerebral ischemia is of great importance. ACPT-I was effective in decreasing cell death by 24–26% in mouse cortical neurons (with a 32–40% decrease in LDH) even when administered 30 min after OGD [69]. This effect was at least partially mediated by a decrease in glutamate release by about 44–66% of control conditions when ACPT-I was given before and after OGD, and by 37–49% when given only after OGD [69]. Similarly, the activation of group III mGluRs by ACPT-I in rats subjected to ischemia decreased the infarct size to about 25 to 37%, regardless of whether the compound was administered 30 min after artery occlusion or 30 min after reperfusion. Additionally, ACPT-I also improved physiological parameters in behavioural tests such as stride length, stand and swing duration, and swing speed [69].

Both (7E)-7-hydroxyimino-N-phenyl-1,7a-dihydrocyclopropa[b]chromene-1a-carboxamide (PHCCC) and (1S,2R)-2-[(3,5-dichlorphenyl)carbamoyl]cyclohexane-1-carboxylic acid (VU0155041), positive allosteric modulators (PAMs) of mGluR4, increased by 12% the survival of cortical neurons in OGD conditions (whereas LDH decreased by 16 and 17–20%, respectively). These results clearly indicate that mGluR4 is responsible for the positive effects of the ACPT-I agonist in mouse cortical neurons subjected to OGD [69]. Moreover, the addition of L-AP4 up to 6 h after the induction of anoxia in rat hippocampal neurons showed a significant neuroprotective effect, whereas the treatment 1 h before anoxia increased survival from 30 to 70% [89].

In their review, Sun and co-workers indicated the importance of discerning the acute, lasting up to first 24 h, and post-acute phases after cerebral ischemia, when considering targeting the glutamate receptors. For instance, the application of MK-801, an NMDAR antagonist, 24 h after the ischemic incident did not improve the outcome [90]. On the contrary, the post-acute phase delivery of memantine, also an NMDAR antagonist, showed beneficial effect on brain recovery, including the penumbra region [91].

More reliable in vivo studies on the involvement of group III mGluRs in brain ischemia involve animals with specific comorbidities. Domin and colleagues (2018) performed analogous experiments using ACPT-I on hypertensive rats subjected to 90 min middle cerebral artery occlusion/reperfusion. In this model, the infarct volume decreased by 28.9 and 18%, depending on whether the drug was administered 30 min after the occlusion or 30 min after the reperfusion, respectively [92]. The activation of group III mGluRs by ACPT-I applied 30 min after occlusion markedly improved physiological parameters in behavioural tests, although, the ACPT-I administered 30 min after the reperfusion did not correct all test parameters [92].

These findings highlight the importance of considering comorbidities, such as hypertension, when evaluating the effects of pharmacological interventions in animal models of cerebral ischemia. The differential outcomes observed with the timing of ACPT-I administration suggest that the therapeutic window and specific mechanisms of action may be critical in determining the efficacy of targeting group III mGluRs in the context of ischemic brain injury.

Indeed, mGluR4 appears to be one of the most important group III mGluRs in the response to ischemic brain damage. Permanent middle cerebral artery occlusion in mice lacking the gene for mGluR4 caused a 25–30% increase in infarct volume. Conversely, the injection of PHCCC, a PAM of mGluR4, into rats with transient focal ischemia (induced by endothelin-1) and into mice with permanent middle cerebral artery occlusion, reduced infarct volume by 35–45%, particularly in the caudate-putamen [93]. The animals treated with PHCCC also showed improvements in behavioural tests, such as posture/hang reflex, forelimb/hindlimb placing, limb-use asymmetry, and adhesive tape removal. Importantly, unlike (RS)-PPG, PHCCC was not toxic even if administered in a dose of more than 1 g per kg of body weight for 15 consecutive days [93].

The substantial role of mGluR4 was also revealed in experiments on mouse and rat cerebellar slices. The use of a photoswitchable negative allosteric modulator (NAM) of mGluR4, trans-OptoGluNAM4.1, and a general group III antagonist, MSOP, reduced the parallel fibre-evoked excitatory postsynaptic current (PF-EPSC) amplitude by 29 and 7%, respectively, compared to a 50% reduction with L-AP4 alone. Both trans-OptoGluNAM4.1 and MSOP also diminished the effect of L-AP4 on paired-pulse ratio (PPR), indicative of a presynaptic action of mGluR4. Notably, these phenomena were not observed in slices retrieved from mice lacking the mGluR4 gene [94]. The activity of mGluR4 was also confirmed during the first 10 min of OGD in cerebellar slices, where OptoGluNAM4.1 and MSOP lessened the PF-EPSC reduction by 14 and 18%, respectively, and delayed the OGD-induced depression by 2.1 min [94].

The mGluR7 receptor also appears to be important in neuroprotection against ischemia, similar to mGluR4. The application of AMN082, a potent allosteric agonist of mGluR7, into mouse cerebral neuronal cell culture subjected to OGD improved cell survival by 18–26% (with a 30–42% decrease in LDH release) [79]. Remarkably, the drug was even effective when administered 30 min after the end of OGD, improving survival by 22–25% (with a 34–38% reduction in LDH). The authors established that calpain was the main mechanism of action for mGluR7 in the mouse cortical model of OGD [79].

These findings highlight the significant role of both mGluR4 and mGluR7 in mediating neuroprotective effects against ischemic insults. The ability of mGluR7 agonists to confer protection even when administered post-ischemia suggests that targeting this receptor may be a promising therapeutic approach for mitigating the damaging consequences of cerebral ischemia. The elucidation of the specific signalling pathways, such as the one involving calpain, provides valuable insights into the underlying mechanisms by which group III mGluRs can exert their beneficial effects in the context of ischemic brain injury.

The involvement of group III mGluRs in hypoxia-ischemia occurring in neonates has been scarcely investigated. In the paper, which focused on group II mGluRs, additional information appeared concerning the lack of effect for the group III mGluR and general mGluR agonists, L-AP4 and t-ACPD, respectively, in improving unilateral hypoxia-ischemia induced in 7-day-old rat pups. However, these compounds did cause the blockage of the surge in cAMP [95].

More recently, Wang and colleagues (2023) demonstrated that the expression of mGluR4, mGluR7, and mGluR8, among other ionotropic and metabotropic glutamate receptors, declines in the infarct area of neonatal rats. The decreases of mGluR7 and mGluR8 expression were statistically significant, showing reduction of around 40 and 50%, respectively. This phenomenon was closely linked to a 60% decline in the expression of the transcription factor NRF1. Consequently, overexpression of NRF1 led to improvement in the survival of OGD-treated primary cortical neurons isolated from neonatal rats by about 35%, as well as reduced infarct volume and improved cognitive function, as measured in tests of escape latency and platform passes in a maize. Additionally, NRF1 overexpression delayed glutamate clearance in the intracellular space by 50% [96]. Unfortunately, the quantitative involvement of particular ionotropic and metabotropic glutamate receptors cannot be elucidated from the data provided by the authors.

The presented results suggest that neuroprotective mechanisms involving group III mGluRs may be different or less pronounced in the neonatal brain compared to adult models of ischemic injury. The role of transcriptional regulators, such as NRF1, in modulating glutamate receptor expression and function is worth further investigation in the context of neonatal hypoxia-ischemia.

## Synergistic modulation of group III metabotropic glutamate receptor activity

The combinatorial modulation of group III mGluRs demonstrates their synergistic potential. The research primarily involved the use of PHCCC, an allosteric agonist of group III mGluRs. The use of 1 µM concentrations of PHCCC and VU0155041, a PAM for mGluR4, together with the agonist ACPT-I, resulted in a 17–18% improvement in the viability of primary mouse cortical neurons (with a 15–23% reduction in LDH release) [69]. Worth mentioning, PHCCC and ACPT-I also exhibited synergistic antidepressant-like activity in rats [97].

The study on the animal Parkinson’s disease model revealed the synergistic action of PHCCC and AMN082, a PAM for mGluR7, in combination with L-AP4, at otherwise ineffective concentrations [98]. The synergistic potential can also involve mGluRs from other groups or even ionotropic glutamate receptors, as shown in the case of PHCCC and 7,7a-dihydro-7-(hydroxyimino)-benzo[b]cyclopropa[e]pyran-1a(1H)-carboxylic acid ethyl ester (CPCCOEt), a potent antagonist of mGluR1 [99], or L-2-Amino-3-phosphonopropionic acid (AP-3), a non-specific group I mGluRs antagonist, and MK-801, an antagonist of NMDAR, which when applied together, slightly reduced cell mortality to 12% (being 18 and 40% when applied alone) in rat primary cerebellar granule cells subjected to OGD [100].

In contrast, Jantas and colleagues (2015) did not observe any synergistic action of activating group III mGluRs with ACPT-I and AZ12216052, a PAM for mGluR8, in neither non-effective nor effective concentrations, in the survival of staurosporine-treated SH-SY5Y cells [101].

These findings highlight the complex and context-dependent nature of the interactions between different glutamate receptor subtypes and their modulators. The synergistic effects observed in some studies suggest that combinatorial approaches targeting multiple mGluR subtypes may be a promising strategy for neuroprotection in ischemic conditions. However, the lack of synergy in other models underscores the need for further investigation to fully elucidate the optimal pharmacological interventions.

## General considerations

Targeting ionotropic glutamate receptors as potential drugs for addiction or other neurological disorders has proven challenging due to the significant side effects associated with their modulation. Ionotropic glutamate receptors, such as NMDARs and AMPARs, mediate fast excitatory neurotransmission and play crucial roles in various physiological processes, and were proven to be effective against brain ischemia in laboratory experiments [4, 102–105]. While antagonists of these receptors have shown efficacy in preclinical models of addiction and epilepsy, they often exhibit severe side effects when tested in humans, including psychotomimetic effects, cognitive impairment, and neuronal toxicity [102, 106–114]. This is because these receptors are widely distributed throughout the brain and are involved in essential functions such as learning, memory, and neuronal plasticity. Blocking them indiscriminately can lead to widespread disruption of normal brain activity, resulting in adverse effects that outweigh the potential therapeutic benefits.

The ubiquitous distribution and engagement of ionotropic glutamate receptors in fundamental neuronal processes make it difficult to target them selectively without causing unintended consequences. Indiscriminate modulation of these receptors can have far-reaching effects on brain function, leading to undesirable side effects that have hampered the clinical development of drugs targeting these receptors for the treatment of addiction, epilepsy, and other neurological disorders. This challenge has prompted researchers to explore alternative approaches, such as targeting metabotropic glutamate receptors (mGluRs), which offer the potential for more selective and nuanced modulation of glutamatergic signalling. The diverse subtypes and distinct localization of mGluRs within the brain may provide opportunities for developing therapeutic interventions with improved safety and efficacy profiles compared to direct targeting of ionotropic glutamate receptors.

When considering the involvement of mGluRs in the pathophysiology of glutamate transmission, including cerebral ischemia, it is crucial to account for the differences between neonatal and adult brains, as well as the differences between various experimental models. The work of Saransaari and Oja (2000) provided interesting insights on this subject, using taurine release as an indicator of glutamate leakage in hippocampal slices from 6-8-day and 3-month-old mice [115]. Even in control conditions, the efflux rate of taurine was much more profound, about 23-fold higher, in hippocampal slices from adult animals compared to neonates. Interestingly, the activation of group II mGluRs by DCG-IV resulted in almost 10-fold increase in efflux rate, but only in hippocampal slices derived from neonates, while slightly decreasing the efflux rate in slices from adult mice. Surprisingly, the group III mGluR agonist L-AP4 or the antagonist (RS)-α-cyclopropyl-4-phosphonophenylglycine (CPPG) caused a slight inhibition of the efflux rate in slices from neonatal rats, with the effect being true only for L-AP4 in slices from adult animals. Conversely, the general AMPARs, kainate receptors, and group I mGluRs agonist, quisqualate, augmented the efflux rate by about 5-fold in slices from neonates, with no effect in slices from adult rats [115]. However, the authors tested all selected compounds at the same concentration (100 µM), regardless of their different affinities for the receptors, which limits the informative value of direct comparisons.

The developmental stage should be also taken into account even when planning in vitro experiments. The sensitivity of neuronal cells additionally depends on the specific EAA used. Cerebral cortex neurons became sensitive to glutamate in the 4th day of culture, whereas the cerebellar granule cells were much more resistant, with ED_50_ value of 70 µM, and glutamate toxicity manifested only from day 8 of in vitro culture [72]. The general mGluRs agonist, (1S,3R)-1-aminocyclopentane-1,3-dicarboxylic acid ((1S,3R)-ACPD), was found to prevent the apoptosis of rat cerebellar granule cells grown in low K^+^ medium after the 5th day, coupled with a substantial decline in the activity of the polyphosphoinositide hydrolysis-coupled mGluRs and the developmentally controlled apoptosis occurring in the developing cerebellum [116–118]. On the other hand, when older cultures of granule cells, at 7-8th day, were subjected to low K + conditions, the L-AP4 and L-SOP prevented apoptosis, with CPPG, a specific mGluR4 antagonist, diminishing their beneficial effect. This phenomenon, together with the decline in the expression of the mRNA of this receptor, clearly indicates the involvement of specific group III mGluR at the later developmental stage [118]. The neuroprotective effect of group III mGluRs was confirmed in cerebellar granule cells treated with β-amyloid peptide [119].

These findings highlight the complex and age-dependent roles of different mGluR subtypes in the regulation of excitotoxicity and cell death pathways in the neonatal versus adult brain. Further research is needed to elucidate the precise mechanisms underlying these developmental differences and their implications for therapeutic interventions targeting mGluRs.

The wide range of research models used, including primary neuronal cell cultures, selected brain slices, and animal studies with varying ages, makes the assessment of the involvement of different mGluRs in brain ischemia quite complicated. For example, it has been shown that in the rat substantia nigra pars compacta, only the mGluR4 receptor was responsible for the attenuation of excitatory transmission. In contrast, in the mouse substantia nigra pars compacta, both mGluR4 and mGluR8 were involved in this process [120]. The differential involvement of mGluR4 and mGluR8 in the substantia nigra pars compacta between rats and mice underscores the need for caution when extrapolating results from one model system to another. Importantly, it is crucial to take into account the cell types used in the experiments and the spatiotemporal distribution of investigated proteins. Although, it is widely accepted that group III mGluRs can activate GIRKs, it was shown in heterologous setup in *Xenopus levis* oocytes or HEK293 cells [121–123]. Considering the mainly presynaptic expression of group III mGluRs (excluding mGluR6) and chiefly postsynaptic and dendritic localization and physiological role [124–128] as compared to sporadic and specialized presynaptic presence of GIRKs [129–131], it seems little probable that this receptors could modulate the activity of the latter in physiological conditions. As indicated, only the mGluR4 played a role in neuroprotection of cerebellar granule cells in later developmental stage [118]. On the other hand, it seems plausible that, in cortical neurons, the activation of both group II and group III mGluRs can be beneficial in neurotoxic conditions since the application of (1S,3R)-ACPD, DCG-IV, L-AP4, or L-SOP, all improved the survival of β-amyloid treated cells [119].

One of the reasons for the difficulties in comparing and interpreting the results on the involvement of mGluRs in the pathophysiology of brain ischemia, including the neonatal form, could be the subtle differences in extracellular and/or intracellular microenvironmental conditions. The extracellular fluid in the brain can be significantly acidified after excitotoxic brain damage. For example, the extracellular pH in the ischemic hemisphere decreased by about 0.81 in fasted hypertensive rats 1 h after the occlusion of the right middle cerebral and common carotid arteries. When the rats were given glucose, the pH lowered even more substantially, by 1.36. Meanwhile, the intracellular pH dropped by only 0.15, with no visible influence when rats were fed with glucose [132].

Indeed, the mGluR4a subtype was completely unresponsive to L-glutamate or (2 S,1’S,2’S)-2-(carboxycyclopropyl)glycine (L-CCG-I), a non-specific group II mGluRs agonist, at pH 6.5, in contrast to the mGluR1a, mGluR5d, and mGluR8b subtypes, which were also included in the study [133]. Considering the fact that the extracellular pH is greatly impacted by hypoxia-ischemia, it would be valuable to know the specific extracellular pH value in every experiment elucidating the role of mGluRs during this pathophysiology. This information could help explain the varying results observed across different studies and model systems, as the sensitivity of mGluR subtypes to changes in pH may play a crucial role in their functional responses.

When investigating the role of metabotropic glutamate receptors in brain ischemia, it is important to remember that other neural cell types, such as glia, including oligodendrocytes, can also express these receptors. Additionally, this expression can be developmentally regulated and can influence the vulnerability of the cells to hypoxic-ischemic conditions. For example, the activation of both group I and group II mGluRs (most probably mGluR3) restored the survival of OGD-treated oligodendrocytes from the optic nerve up to 75–85% of normoxic conditions when the cells were retrieved from neonatal rats (P8-12). In contrast, only group I mGluRs were still effective in young adult rats (P30-35) [134]. Notably, the postnatal optic nerve oligodendrocytes were far more sensitive to OGD than the cells from young adult rats, leading to 70% mortality compared to 30%, respectively [134].

Additionally, glial cells can influence the survival of neurons. When group II or III mGluRs were activated in rat primary astroglial cells treated with lipopolysaccharide, the conditioned medium collected from these cells increased the survival of rat primary mesencephalic neurons up to 80–85% of control conditions [135]. Interestingly, both astrocytes and oligodendrocytes can produce brain-derived neurotrophic factor (BDNF) upon the activation of mGluRs [136, 137].

The complex interplay between different neural cell types and the developmental regulation of mGluRs expression and function is worth consideration. The ability of glial cells to modulate neuronal survival through mGluR-mediated mechanisms suggests that targeting these receptors in non-neuronal cells may be an important consideration in developing therapeutic strategies for ischemic brain injury.

It is important to emphasize that within cells, whether neurons or glia, glutamate receptors interact with each other, and this regulation can differ between neonates and adults. One of the main clues to these interactions can be derived from the common co-localization of mGluRs in different brain regions. For instance, in the neonatal rat cortex, the response triggered by group I mGluRs was augmented by group II receptors [138]. This suggests that the functional interplay between mGluR subtypes is an important factor in determining the overall cellular response to glutamatergic signalling and that this interplay may be developmentally regulated.

The complex interactions between different glutamate receptor types, both ionotropic and metabotropic, as well as their differential expression and regulation during brain maturation, underscore the challenges in elucidating the precise roles of mGluRs in the pathophysiology of ischemic brain injury. Accounting for these multi-layered interactions and developmental differences is crucial for a comprehensive understanding of the therapeutic potential of targeting specific mGluR subtypes in the context of neonatal and adult cerebral ischemia.

## Challenges and future directions

Despite the significant progress in understanding the functions of group III mGluRs, several challenges remain in the development of effective therapeutic interventions targeting these receptors. The complex and sometimes opposing roles of different group III mGluR subtypes in various brain regions and pathological conditions necessitate a deeper understanding of their precise mechanisms of action and the development of highly selective pharmacological tools.

For example, while activation of mGluR4 has been shown to have neuroprotective effects in models of Parkinson’s disease, the role of other group III mGluR subtypes, such as mGluR7 and mGluR8, in the same pathological context is less clear [139–141]. Elucidating the specific contributions of each subtype to the modulation of neuronal excitability, synaptic transmission, and neuroplasticity in different brain regions and disease states will be crucial for the rational design of subtype-selective compounds.

Additionally, the potential for receptor desensitization and the development of tolerance to group III mGluR modulators over prolonged treatment periods require further investigation [142]. Chronic activation of these receptors may lead to adaptive changes in receptor expression, signaling pathways, and downstream effectors, which could limit the long-term efficacy of pharmacological interventions. Strategies to mitigate receptor desensitization, such as the development of biased agonists or allosteric modulators, may help overcome this challenge [142, 143].

Future research should also explore novel approaches to targeting group III mGluRs, such as the use of bivalent ligands or bitopic compounds that can engage multiple receptor subtypes or binding sites simultaneously [143]. This could potentially enhance the selectivity and potency of group III mGluR modulators, leading to more effective and safer therapeutic interventions.

Moreover, the integration of group III mGluR modulation with other pharmacological or non-pharmacological interventions, such as hyperbaric oxygen therapy, neuromodulation techniques or lifestyle modifications, may provide synergistic benefits and improve the overall therapeutic outcomes for patients suffering from neurological and psychiatric disorders, including brain ischemia [144].

## Data Availability

No datasets were generated or analysed during the current study.

## Abbreviations

(1S,3R)-ACPD: (1S,3R)-1-aminocyclopentane-1,3-dicarboxylic acid, a non-specific agonist of mGluRs
t-ACPD: (+/-)-1-aminocyclopentane-*trans*-1,3-dicarboxylic acid, an equimolar mixture of two enantiomers, (1S,3R)-ACPD and (1R,3S)-ACPD
ACPT-I: (1S,3R,4S)-1-aminocyclopentane-1,3,4-tricarboxylic acid, a non-specific agonist of group III mGluRs
AIDA: (RS)-1-aminoindan-1,5-dicarboxylic acid, a non-specific antagonist of group I mGluRs
AMN082: N,N′-dibenzhydrylethane-1,2-diamine, a specific allosteric agonist of mGluR7
AMPAR: α-amino-3-hydroxy-5-methyl-4-isoxazolepropionic acid receptor
cAMP: cyclic adenosine monophosphate
L-AP3: L-2-amino-3-phosphonopropionic acid, a non-specific antagonist of group I mGluRs
L-AP4: L-2-amino-4-phosphonobutyric acid, a non-specific agonist of group III mGluRs
AZ12216052: 2-[[(4-bromophenyl)methyl]thio]-N-[4-(1-methylpropyl)phenyl]-acetamide, a specific positive allosteric modulator of mGluR8
L-CCG-I: (2S,1’S,2’S)-2-(carboxycyclopropyl)glycine, a non-specific agonist of group II mGluRs
CPCCOEt: 7,7a-dihydro-7-(hydroxyimino)-benzo[b]cyclopropa[e]pyran-1a(1H)-carboxylic acid ethyl ester, a selective non-competitive antagonist of mGluR1
CPPG: (RS)-α-cyclopropyl-4-phosphonophenylglycine, a specific antagonist of group III mGluRs
DCG-IV: (2S,1’R,2’R,3’R)-2-(2,3-dicarboxycyclopropyl)glycine, a selective agonist of group II mGluRs
DHPG: (S)-3,5-dihydroxyphenylglycine, a non-specific agonist of group I mGluRs
EC_50_: half maximal effective concentration
GIRK: G-protein-coupled inwardly-rectifying potassium channel
KA: kainate
LDH: lactate dehydrogenase
LTP: long-term potentiation
mGluR: metabotropic glutamate receptor
MK-801: (5S,10R)-5-methyl-10,11-dihydro-5H-dibenzo[a,d]cyclohepten-5,10-imine hydrogen maleate, dizocilpine, an antagonist of NMDAR
MSOP: (RS)-α-methylserine-O-phosphate, a non-specific antagonist of group III mGluRs
NAM: negative allosteric modulator
NMDAR: N-methyl-D-aspartate receptor
OGD: oxygen-glucose deprivation
PAM: positive allosteric modulator
PF-EPSC: parallel fibre-evoked excitatory postsynaptic current
PHCCC: (7E)-7-hydroxyimino-N-phenyl-1,7a-dihydrocyclopropa[b]chromene-1a-carboxamide, specific positive allosteric modulator of mGluR4
PKA: protein kinase A
(RS)-PPG: (RS)-4-phosphonophenylglycine, a non-specific agonist of group III mGluRs
PPR: paired-pulse ratio
L-SOP: L-serine-O-phosphate, a non-specific agonist of group III mGluRs
VU0155041: (1S,2R)-2-[(3,5-dichlorphenyl)carbamoyl]cyclohexane-1-carboxylic acid, a specific positive allosteric modulator of mGluR4
